# Human resources for maternal health: multi-purpose or specialists?

**DOI:** 10.1186/1478-4491-6-21

**Published:** 2008-09-30

**Authors:** Vincent Fauveau, Della R Sherratt, Luc de Bernis

**Affiliations:** 1 Technical Services Division, UNFPA (Geneva Office), 11 Chemin des Anemones, 1219 Chatelaine, Switzerland; 2 Wotton under Edge, UK; 3 Africa Division, UNFPA, Addis Ababa, Ethiopia

## Abstract

A crucial question in the aim to attain MDG5 is whether it can be achieved faster with the scaling up of multi-purpose health workers operating in the community or with the scaling up of professional skilled birth attendants working in health facilities. Most advisers concerned with maternal mortality reduction concur to promote births in facilities with professional attendants as the ultimate strategy. The evidence, however, is scarce on what it takes to progress in this path, and on the 'interim solutions' for situations where the majority of women still deliver at home. These questions are particularly relevant as we have reached the twentieth anniversary of the safe motherhood initiative without much progress made.

In this paper we review the current situation of human resources for maternal health as well as the problems that they face. We propose seven key areas of work that must be addressed when planning for scaling up human resources for maternal health in light of MDG5, and finally we indicate some advances recently made in selected countries and the lessons learned from these experiences. Whilst the focus of this paper is on maternal health, it is acknowledged that the interventions to reduce maternal mortality will also contribute to significantly reducing newborn mortality.

Addressing each of the seven key areas of work – recommended by the first International Forum on 'Midwifery in the Community', Tunis, December 2006 – is essential for the success of any MDG5 programme.

We hypothesize that a great deal of the stagnation of maternal health programmes has been the result of confusion and careless choices in scaling up between a limited number of truly skilled birth attendants and large quantities of multi-purpose workers with short training, fewer skills, limited authority and no career pathways. We conclude from the lessons learnt that no significant progress in maternal mortality reduction can be achieved without a strong political decision to empower midwives and others with midwifery skills, and a substantial strengthening of health systems with a focus on quality of care rather than on numbers, to give them the means to respond to the challenge.

## Background

As the international public health community marks the twentieth anniversary of the Safe Motherhood Initiative [1], more than 530 000 women still die each year from complications of pregnancy and childbirth, over 90% of them in South Asia and sub-Saharan Africa. Additionally, 10 to 20 million women annually suffer severe health problems as a result of pregnancy and childbirth, such as obstetric fistula or chronic infection. Seventy percent of maternal deaths are due to five major complications, the majority of which occur during labour, delivery and the post partum period. Approximately 15% of women will experience a complication during pregnancy, childbirth or the immediate postpartum period – most of which cannot be predicted, but almost all of which can be managed. Most maternal death and disability could be averted if:

• all pregnancies were wanted,

• all births were attended by skilled health professionals and

• all complications were managed in quality referral facilities offering emergency obstetric care [2].

While the focus of this paper is on the second of these conditions, it must not be forgotten that a large part of maternal deaths could be avoided if all women had access to family planning and reproductive health services. It must also be acknowledged that the interventions to reduce maternal death also significantly contribute to reducing newborn mortality.

Saving mothers' lives is widely recognized as an imperative for social and economic development, as well as a human rights imperative, although until recently there has been limited evidence mapping such links[3]. It is the basic right of every woman and baby to have the best available care to enable them to survive pregnancy and childbirth in good health. Yet, while the techniques and strategies to address maternal health are well known and widely accepted, and the need for access to specialist emergency obstetric care services has a high level of evidence [4], the factor most neglected in the last decade was human resources required to implement these interventions. Although there is a general consensus that maternal mortality and morbidity cannot be reduced without midwives and others with midwifery skills, the numbers of these skilled providers have not significantly increased over the last two decades. Moreover, the actual numbers of skilled midwifery providers has started to decrease in some countries, as the result of migration, losses from HIV/AIDS and dissatisfaction with remuneration and working conditions. At the same time issues of quality of care remain crucial, particularly where health systems do not play their supportive role, as in many countries that have embarked in scaling up the number of community-based providers without giving sufficient attention to their skills. The World Bank estimates that maternal deaths would decrease by 73% if coverage of key interventions rose to 99% [5]. Access to essential maternal health care services, however, is riddled with inequities. The lower a woman's economic status, the less likely she is to have skilled assistance at delivery and lifesaving emergency obstetric care [2]. Geographical location, ethnicity and age are also related to disparities in access.

WHO initiated a decade of special attention to the health workforce with the World Health Report 2006, 'Working together for Health'[6]. UNFPA, working jointly with the International Confederation of Midwives (ICM), plans to contribute to this global initiative on the health workforce by initiating in collaboration with their partners a global campaign to promote and rapidly scale-up the coverage of midwifery care. Midwives and others with midwifery skills are the representation of UNFPA's mandate within the health workforce, not only for their role in providing skilled delivery care, but also for their ability to deliver the essential sexual and reproductive health package in relation to maternal health. In addition, efforts to strengthen midwifery are also in line with UNFPA's mandate to promote gender equality, as midwives are key female members of the health workforce. However, for many reasons, some having to do with the fact that most midwives are women, there has been gross underinvestment, and sometimes no investment at all, in building or maintaining a cadre of professional midwives. In addition, midwives very often have low status within their community and receive little recognition. The vast majority of midwives thus suffer from the same gender-related inequalities as other women. The result has been insufficient investment in midwifery training, deployment and supervision, coupled with inadequate regulation and policies to support and protect midwives in their practice. Yet, without expert midwives to teach midwifery skills and supervise others, ensuring quality of care will not be possible and efforts to reduce maternal and newborn deaths will fail. A number of countries or states – particularly Sri Lanka, Malaysia, Tunisia, Thailand, Kerala, Tamil Nadu – have, however, successfully undertaken specific measures to make midwifery a respectful and attractive profession. Policy, advocacy and revision of regulatory systems were instrumental in order to professionalize midwifery and remove discriminatory legislation.

The Millennium Development Goal 5 highlights the crucial role of midwives and others with midwifery skills on the path to improved maternal health by including as its second indicator the proportion of births attended by skilled health providers. Although the percentages are not specified, it is assumed that the target for 2015, "universal access to a skilled birth attendant", translates into between 90% and 100% coverage. Currently it is estimated that no more than 40% of births in low-income countries are assisted by properly skilled attendants – highlighting the large effort needed to reach the target of 90% coverage by 2015 [7]. According to WHO [2], an additional 334 000 midwives are required to fill this gap, not counting the number of doctors and other nurse providers. It can be argued that at least twice as many are required to achieve universal access to a full package of sexual and reproductive health care.

In the past few years, the international public health community has made two significant advances. One by incorporating in to the new global health partnerships the health care professional organizations such as the International Confederation of Midwives (ICM) and the International Federation of Gynecology and Obstetrics (FIGO). The other by highlighting the key role of human resources for health (HRH) in the failure of health systems and the need to address HRH in priority in health system strengthening initiatives (GAVI-HSS, GFATM, Global Business Plan, Global Campaign for Health MDGs, International Health Partnership, etc).

This paper aims at contributing to generating a massive effort to increase not only the coverage of all births by skilled attendants, but also the quality of this attendance by promoting the role of midwives and others with midwifery skills in improving maternal, reproductive and newborn health. The question, however, is whether countries should give priority to producing a relatively high number of multipurpose community-based providers to cover all villages or to produce a lower number of specialized, facility-based, professional and skilled maternal health providers [8].

### Situation and challenges

Ensuring equitable access to a continuum of skilled care before, during and after childbirth, is recognized as a universal human right, and is critical for saving the lives of mothers and for their newborns [2,9-11]. However, skilled care requires skilled providers – a scarce commodity in most low-income countries. Much of the efforts in the lead up to the 20 year marking of the Safe Motherhood Initiative (SMI), have focused on the barriers to skilled care are at birth, among which the lack of qualified human resources appears the most challenging.

The lack of skilled providers linked to a facility offering quality emergency obstetric and neonatal care (EmONC), is neither a new phenomena, nor is it only a problem of low-income countries. The need to invest in training of the midwifery workforce and ensuring that midwifery providers have appropriate life-saving skills have been topics of debate for many decades [12,13]. Yet, as estimates for the proportion of births attended by a skilled provider shows, the majority of women in developing countries still give birth without such assistance and the data reveals huge disparities and inequity, with women in low income families having little options or opportunities to access such healthcare [2,7]. However, the lack of access to health services occurs for a variety of reasons and not just because of lack of healthcare providers [14].

A 'skilled birth attendant' (SBA) has been defined by the WHO in collaboration with the ICM and FIGO and has been endorsed by UNFPA, the World Bank and the International Council of Nurses in 2004 [15]. The definition builds on and seeks to add clarity to the initial definition in the 1999 Joint statement on Maternal Mortality [16] and the one developed by the Interagency Group for Safe Motherhood in 2000 [17], and sets better the minimal requirement for a skilled birth attendant.

The 2004 definition states that a skilled birth attendant is: "an accredited health professional – such as a midwife, doctor or nurse – who has been educated and trained to proficiency in the skills needed to manage normal (uncomplicated) pregnancies, childbirth and the immediate postnatal period, and in the identification, management and referral of complications in women and newborns." [15]

As the above definition clearly shows SBAs are not a single cadre or professional group. SBAs are providers with specific midwifery competencies; they perform these competencies as professional midwives or, if trained in these competencies as general practitioners with midwifery competencies, or as nurses. Furthermore, not only must they have received proper training to carry out their tasks, but they must have developed the competencies to a level of proficiency. The total list of competencies for each type of skilled attendant will vary between the different professional groups, according to the scope of practice for each group. The list may even vary for cadres with same professional title in different countries, depending on the legislation and regulations and training curricula for each cadre. The common denominator, however, is the basic skills required to assist a woman during pregnancy, childbirth and after birth, including essential care to newborns – known internationally as 'midwifery skills' and defined as "core competencies". In addition, experts agree that the education of nurses and midwives must include development of problem-solving competencies, because the arrival of a woman at a referral facility is often the end of a long and complex decision-making process, influenced by the interpersonal relationships between the woman, her family members and the health providers. [18]

Moreover it is known that to be effective, healthcare providers must work in a supportive enabling environment – which must include basic equipment and drugs as well as good communication and transportation systems – to ensure timely referrals when needed and have effective and supportive supervision. Yet, too often, the enabling and supportive environment is also lacking.

### Midwifery skills

The 'core competencies' required of any skilled birth attendant outlined in the 2004 WHO ICM FIGO statement were intended to apply to any health worker providing midwifery care at any level of the healthcare system, including the primary care level. Included within the core competencies are the basic EmOC skills to which essential neonatal care has been added, as well as essential maternal and neonatal healthcare for preventive and promotional care and care of women and newborn with no complications. The list of 'additional competencies' was added in the 2004 statement to apply to those skilled birth attendants working in peripheral and or isolated settings, where referral to a district hospital is difficult. Whereas the 'advanced skills' are the surgical competencies required for comprehensive care (EmONC).

Contention however remains as to which maternal health providers should have these core competencies. Is it all maternal health care providers? And, who should have just the core and who should have advanced or additional competencies? Moreover, the discussion on which maternal health workers can be trained or 'up-skilled', to ensure they have the required competencies to a level of proficiency, is causing concern in many countries.

Even if there was a consensus on the above questions, there remains the issue of the maintenance of these competencies. And the issue of whether the legal and regulatory framework properly protects the rights of the healthcare provider to perform the life-saving interventions for maternal and newborn survival. Often they are seen as the prerogative only of physicians. Therefore, becoming competent, or scaling up the competencies of the maternity workforce, is only part of the overall issue to be addressed. To develop and implement a plan for the adequate production of their maternity workforce, the countries need to know how many of which type are needed, where they should be deployed, and how to retain them at their post, especially those working in rural areas.

### Why have the critical midwifery competencies been so neglected?

One of the major reasons explaining why so many countries still have inadequate numbers of skilled midwifery providers is because those grappling with human resources have not paid attention to the need for 'proficiency' in the various competencies required to assist women and newborns. For too long it has been accepted that as long as the health worker received some (often too little) training in midwifery, this was sufficient. Too often there has been a lack of understanding and appreciation of the difference between competence – the ability to carry out a task to the required standard – and competencies, the discreet knowledge, skills, attitudes and experience required for individuals to perform their jobs correctly and proficiently [19].

Additional reasons for the current shortfall in midwifery skills in many low-income countries include the lack of understanding and appreciation of what the professional midwife can offer, as well as an historical prioritisation on medical training of physicians over other healthcare providers. As argued in the World Health Report 2005, many countries facing current shortages of midwifery providers have been at the mercy of misguided, albeit well intentioned, advice from external donors recommending policy changes to create a multipurpose worker [2,20] or seeing midwifery care as a voluntary occupation that can be performed by a traditional healer or traditional birth attendant.

Investing in a specialist midwifery provider is challenging in many countries because midwifery, as a predominantly female profession, does what is predominantly considered 'women's work' [21]. The double burden of being a woman, herself subject to gender inequalities, as well as being a female worker, puts tremendous pressure on midwives who do a very emotional and stressful job that can lead to high levels of occupational 'burn-out' [22-24]. Having responsibilities for their own home and child care, etc., and working with women in what some perceive as a female area – pregnancy and birth – is made even more difficult in those situations where women's status is low and where assisting childbirth is seen as low status or culturally unclean. On a positive note however, where midwives are respected they can, by working in the community, in close proximity to families, have the potential for offering career aspirations to girls and young woman and in so doing, may contribute to efforts to address gender inequity. [21]

The failure of governments to provide competent, skilled midwifery health workers has been seen by some as a blatant case of gender inequality or lack of gender sensitive health policy [25]. Failure of governments to provide basic healthcare for the most vulnerable of its citizens at the most vulnerable time of life can be viewed in the light of the Committee on Economic, Social and Cultural Rights' General Comment 14 as a failure of governance [26].

### Why invest in midwives and others with midwifery skills?

Investing in a specialist cadre of midwifery provider-professional midwives or others with midwifery skills – has been shown to make a difference in reducing maternal mortality in many countries. Indeed, historical evidence tells us that the countries that have succeeded in reducing their maternal mortality and morbidity have done so by ensuring skilled care at ALL births [8,27-29]. In particular, they have achieved this by ensuring that all home births were undertaken by 'trained and supervised midwives or, as in the case in Sweden and the UK, by making sure midwives not only referred all complicated cases – having first rendered first aid and offered first line management – but also reported all births and maternal deaths to the local public health physician or district health authority. [30]. Reviewing case studies from countries that have in recent years succeeded in reducing their maternal mortality ratio, Koblinsky suggested that, "assistance at birth by a skilled birth attendant in the home or any health facility, supported by a functioning referral system, can reduce the MMR down to around 50 or below" [28]. The recent Lancet series on maternal survival also point to the value of midwives working as a team in health centres [31]. Indeed, home delivery is not a good use of the time of scarce professionals, who should be concentrated in health centres.

For skilled attendants to effectively contribute to achieving the MDGs however, they must be accessible, offer affordable women-centred care, and must be seen as a member of the health system and to be credible. For this they must be technically competent. Being seen by the community as a specialist in midwifery care contributes to credibility. The outstanding evolutionary feature of maternity-related health services in Sri Lanka and Malaysia is the pivotal role of trained and government employed midwives. They have been relatively inexpensive to both countries, yet they have been the cornerstones for the expansion of an extensive health system to rural communities. They have provided accessible maternity services in hospitals and communities, gained sustained respect from the communities they serve, and are described with affection and admiration by managers and policymakers in each country' [32]. As found in a study on access to emergency obstetric care and human resources in Tanzania, there is a positive correlation between having a professional qualification and clients' willingness to use health services [33].

Professional midwives or others who meet the international definition of a midwife [34] (regardless of their title) and practice according to ICM's evidence-based essential midwifery competencies [35] do have all the essential basic midwifery competencies required for the provision of high quality skilled midwifery care, and more. Where they work in partnership with women and are acceptable by women and their communities, professional midwives (or those functioning with legal protection as a professional midwife) offer countries potential for meeting the broader reproductive health needs of communities [21,36], as well as contributing to universal primary health care for all [37]. As history has shown, midwives can be most useful in helping to ensure that health services reach those in greatest need, the poor and hard to reach communities [38,39].

### Quality or quantity?

While there is a need to build the capacity of the maternity workforce in terms of quantity in order to reach out to all communities, it is even more important to consider quality. The debate on whether to prioritise quality or just have more numbers is at the heart of current discussions on skilled attendants, and strategic decisions are likely to have a strong impact on maternal mortality. Whilst everyone agrees it is not effective to look at human resources for health for a specific health issue in isolation [40], we argue that MNH services do have several unique characteristics that require specific attention when making decisions about the size, shape and production of the midwifery workforce. Specifically the need exists for:

• High levels of technical competence in a number of very specific areas, both curative and promotive in nature. Maternal mortality reduction shows the greatest sensitivity to the presence of skilled maternal health providers [41].

• Appropriate curricula that ensure sufficient time for hands-on practical training to become competent to the level of proficiency in all the requisite areas, as complications can arise quickly and without warning. What is required is repeated reflexive and intelligent practice [42,43]. Clinical instruction and mentorship are also paramount. Trainers must themselves be proficient in these competencies, although unfortunately in many low-income countries they are not [44].

• Gender sensitivity. Although this can apply to all health service access [45], lack of a female provider is perceived as one the major barriers to why women do not use maternal health care [46,47].

• Excellent inter-personal communication and cultural competencies, because of the high cultural sensitivity of pregnancy and birth. Nowhere else are interpersonal skills, linguistic skills and cultural appreciation more crucial to help the families with decision making in all aspects of reproductive health [18,46,47].

• Motivation for the job – has been shown to be vital for providing quality care [48-50]. Midwifery providers must be available at all hours of the day and night – whenever birth takes place. Among the criteria that should be considered are demonstrating professionalism and positive attitude to patient, avoiding impersonal routine response, and resisting to corruption [51].

For all the above reasons it is essential that curricula and training programmes prioritise midwifery skills – but sadly many current training programmes do not. Far too often, midwifery skills are seen as accessory, or add-on skills, and are afforded little time, typically at end of a programme, where there is little time for repeated hands-on practice.

**Figure 1:**
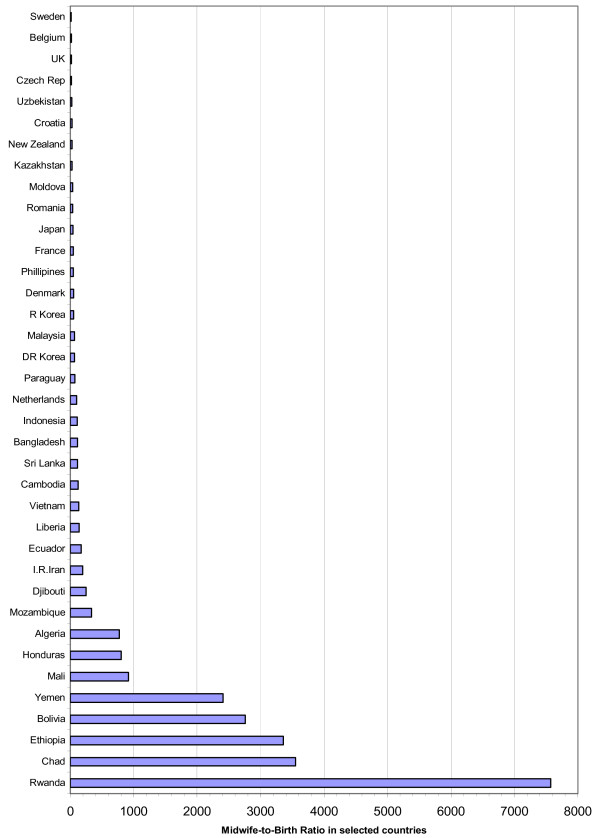
Expected births per midwife ratio in selected countries.

In terms of numbers, the largest barrier to overcome is the need for sufficient teachers and trainers who are competent in education and in midwifery theory and in clinical practice. Deciding on numbers depends on a complex set of criteria: number of training institutions and teachers, caseload, overall education standards, reservoir of suitable entrants, but also recruitment policies, fiscal space and budget. Historically, a population base ratio has been used to estimate the number of midwives needed in a given country. The most widely used ratio of one midwife to 5000 population developed by WHO in 1993 [12], assumes that one community midwife would be able to care for 200 pregnant women a year, including assisting at their births and giving postnatal follow up care. The ratio however does not take account of the skill-mix needed to care for obstetric emergencies, nor the different geographical circumstances, differences in fertility rate nor other personal or professional work demands on the midwife. UNFPA has recently called for using a new "births by midwife" indicator i.e. the number of births expected to be attended in all security by a qualified midwife [36] (see Figure 1).

To achieve the right balance between numbers and quality, adequate funds and a cost-effectiveness analysis are necessary, in turn dependant upon having policies and strategies in place. To avoid repetition of past mistakes and the selection of misguided strategies, technical competence is critical to guide the decision.

### Towards solutions: key areas

Time to scale up is limited. However, as countries like Indonesia have experienced, rapid scale up in numbers without ensuring full competencies of midwifery providers can be costly in terms of in-service training needs [52]. It is also possible to improve access to skilled care by better utilization of existing staff, and training mid-level providers in tasks that are usually undertaken by physicians [53,54]. Each country will need to take a considered approach, allowing fast scale-up while at the same time maintaining, or improving, quality. While there is a need to address the deficiencies in specific obstetric skills, especially surgical skills and specialist neonatal skills, it is the midwife who will ensure access to all. Graham et al estimate that on average there should be a minimum of five midwives for 1 obstetrician (or physician with obstetric skills) [55]. Midwives are also required to develop community capacity in order for communities to take their place in monitoring and evaluating maternity services and contributing to overall quality improvements [47].

Midwives and other midwifery providers perform best within a multi-professional team of health workers – including peers – but also support workers who can conduct some of the non-specialist midwifery tasks under their supervision. Physicians with obstetric skills or mid-level providers with obstetric competencies (such as in selective surgical procedures) are best targeted at referral centres where surgery is possible. This partnership should be based on mutual respect and appreciation for each other's contribution, rather then on an outdated historical hierarchical model, which sees the midwife or other mid-level worker as subservient to the physician.

In addition to training, capacity building and capacity-development require attention to structure, systems, roles, support, supervision, as well as logistics [56]. Above all, any new initiative must have inbuilt from the beginning a robust monitoring and evaluation systems, not only to demonstrate when progress is being made, but also to monitor quality improvement and future decision making that is at the heart of any capacity-development initiative [57].

**Figure 2:**
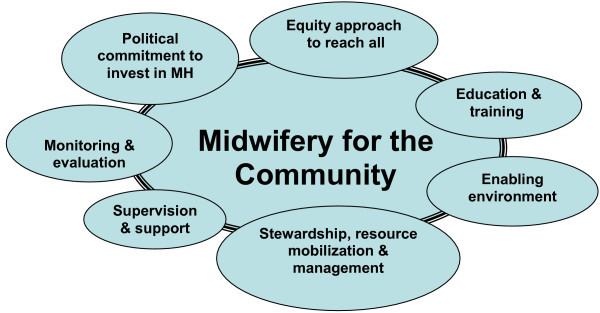
Framework for addressing issues of scaling-up midwifery for the community level.

During the 1^st ^International Forum on midwifery in the community held by UNFPA, ICM and WHO in 2006 [[58] and Additional file 1], a framework was proposed for rapid scale-up of midwifery providers, based on a capacity development model. The framework identifies seven interconnected areas of work (Figure 2):

1. Policy, legal and regulatory frameworks

2. Ensuring equity to reach all

3. Recruitment and education (pre- and in-service), accreditation,

4. Empowerment, supervision and support

5. Enabling environment, systems, community aspects

6. Tracking progress, monitoring and evaluation, numbers and quality

7. Stewardship, resource mobilization

#### 1. Policy, legal and regulatory frameworks

All the above areas of work are interrelated, but political and legislative action must be in the forefront. The protection to which mothers and children are entitled under the right to health framework cannot be regarded as 'charity'. It is an obligation of governments, irrespective of adverse conditions such as severe shortage of economic resources [2,9,21,22,59-61]. While governments cannot be held responsible for the actual care or omissions of care given by individual practitioners, they are responsible for ensuring that adequate mechanisms are in place for regulation, delegation of authority and training of the providers and that appropriate policies are implemented. Legal and regulatory frameworks are also needed to protect midwifery and medical providers.

Action: create a coalition of interested stakeholders, including professional associations, to promote and influence policy changes. Such partnerships should be built on mutual respect and include community participation, for example civil society groups, from the start.

#### 2. Ensuring equity in reaching the poor

In all countries poverty is strongly associated with less access and use of healthcare, including skilled midwifery care at birth [62,63]. Evidence shows that even in relatively low-income groups, women with higher levels of autonomy find it easier to access maternal health services [64]. Furthermore, evidence shows that introduction of formal user fees and demands for payment 'under the table' have a negative influence on utilization of maternal health care services, particularly during childbirth [46,48].

Action: making equity a national cause, in collaboration with and involving from the beginning he wider stakeholder group, such as the other ministries, and civil society, NGOs, faith-based and private healthcare providers, media and parliamentarians.

#### 3. Recruitment and education (pre- and in-service), accreditation

Recruiting from and providing education within the local area can help ensure that service provision is culturally appropriate. Both pre-service and in-service education and training programmes should be based on a competency model, with those who teach midwifery in clinical or classroom settings being themselves competent in midwifery and having undertaken adequate preparation for their role. More work is needed to ensure that pre-service midwifery programmes have a better client-centered basis [51]. Improving quality of care depends on the new graduates' ability to practice their newly acquire skills in the real situation. There is a need to develop or strengthen accreditation systems, including ensuring periodic updating and professional continuing education programmes linked to re-registration or re-licensing.

Action: promote national evidence-based standards for education programmes and institutions, ensuring that they are as important as evidence-based clinical standards and protocols. Incentive schemes may be needed in some situations, to encourage and support recruitment from local communities and/or recruitment from linguistically and culturally diverse communities.

#### 4. Empowerment, supervision and support

The problems associated with getting staff to change their performance based on evidence are widely recognized [65]. Because the majority of women will not encounter a problem during pregnancy, childbirth or after birth, few providers may have hands-on practice of managing complications. Indeed, many midwives working at the community level may never have experienced in their initial training some of the problems and complication that they may meet during their professional career. Providing midwives with supportive supervision which helps build their capacity is essential, more so for those working in isolated practice or small teams in the community. For supervision to build capacity it must go further than assessing records and reviewing case registers. It needs to be supportive, undertaken by clinically competent midwives, allow free and open discussion of clinical practices, and give an opportunity for providers to acknowledge their weaknesses [66]. Supervision should empower midwives, should not focus on just filling in a checklist, and should be performed by provincial or national health offices.

Action: Organize supervision as a separate function from the management of the midwifery service, although linked to it and indeed in some areas supervisors may have responsibility for both. Ensure that supervisors are competent in midwifery and receive in-service and updating training in supervising midwifery practice.

#### 5. Enabling environment, strengthening systems, community aspects

Too often this enabling environment is missing – often due to failures in health system management. For example, frequently the essential drugs for EmONC are not included in the national drugs list. It is now well known that health care practitioners cannot carry out all their tasks and function effectively if they have concern for their own safety or that of their family, or if they are anxious about their own health or the health of their family [6]. Caring for woman and newborns in an environment lacking essential drugs and equipment to save lives if a complication occurs is particularly stressful and de-motivating. Support from the local community and community leaders, and the active participation of men, are also vital to creating an enabling environment, despite the barriers to male participation [67].

Actions: total quality care improvements, quality circles, as well as needs assessments, clinical audits, community surveys, confidential enquiries into maternal deaths, investigations of near-miss cases: all can be used as means of improving quality of care. A continuous supply of essential drugs down to the community level must be assured.

#### 6. Tracking progress, monitoring and evaluation for numbers and quality

Until recently little attention has been paid to the need for permanent monitoring and periodic evaluation of large midwifery programmes. Very few current programmes have built-in evaluation, and there is consequent uncertainty about their health outcomes, and thus their effectiveness. Most safe motherhood programmes rely on fairly standard process indicators such as the UN indicators [68-71] that are most often used for measuring the availability and use obstetric services, but do not take into account quality, which is the product of technical capacity and culturally appropriate response.

In addition, lack of a universal benchmark to define a skilled birth attendant has not only caused confusion and lack of validity around this indicator, but has led to great variations and thus an inability to make comparative judgments on programmes [6]. There are currently few reliable and tested tools to measure the midwifery competencies of healthcare providers, or to compare the performance and utilization of non-specialized midwifery providers against specialist provider [72-74].

Actions: Establish regular monitoring based on routine data collection with an emphasis on quality. Monitoring and evaluation should involve midwives and midwifery providers at the community level, so that midwives and the community members can use the findings. This is particularly important for evaluating training initiatives, where – for pragmatic reasons – descriptive, non-experimental designs calling for before-and-after studies are the only option for assessing effectiveness.

#### 7. Stewardship, resource mobilization

While it is acknowledged that most countries need to take incremental steps towards implementing comprehensive health policies to respond to the needs of all citizens, very few have a well designed systematic plan to achieve this [75]. Forty African countries are currently engaged in developing and implementing their national Road Map for maternal and newborn care. Ensuring equitable midwifery care requires intensified actions and substantial investments, calling for increased funds, and better costing and budgeting [76]. In many countries parliamentarians and senior policy makers are not fully aware of the issues around access to midwifery care at the community level and often fail to understand the complexities involved. Furthermore, studies show that decentralization efforts too often focus on financial and structural reforms and do not take sufficient account of the human resource dimension [77,78].

Actions: Governments must provide sufficient expenditure and proportionate investment of public resources in the maternal health sector, and focus expenditure on rectifying existing imbalances in the provision of health facilities, health workers and health services. This includes ensuring that the privatization of the health sector does not create a threat to the availability, accessibility, non-discrimination, acceptability and quality of maternal and newborn health services. Policy makers must also recognize that, even where safe motherhood programmes are built on increasing access to institutional birth, women and newborns need access to community-based midwifery care ante and post-natally, as women are more likely to seek skilled care for birth if they have access to such care ante-natally [79].

### Lessons learned in countries

The issue of requiring a dedicated skilled provider for maternal and newborn health is gaining momentum in many parts of the world – despite pressures for a generic multipurpose healthcare provider. A survey conducted by WHO in the Africa region showed that among the 31 African countries who responded to the survey (out of 46), only 14 had a HRH policy and plan, an HRH situation analysis and an HRH operational plan [80]. For example, WHO-AFRO is about to publish a set of Midwifery Competencies for Africa, recommended by the Regional Committee in 2005 and developed through a series of consultations with countries. It is hoped that countries will use these competencies as benchmarking for agreeing who meets the definition of a skilled attendant. There are also positive signs to show that the various country Road Maps for maternal and, newborn health are offering important opportunities to integrate human resources issues in the national health plans and national sexual and reproductive health policies. Similarly in other regions there is a renewed interest in developing and supporting the specialist cadre of midwifery provider.

### Creating/promoting a specialist midwifery cadre

There are more examples of countries investing in increasing the numbers of multi-purpose maternal health providers, but some countries are also taking steps to strengthen and skill up their current midwifery providers, and/or creating a specialist cadre in an attempt to upgrade quality of obstetric care. For example, action has begun to re-establish midwifery in the south of Sudan, an area of huge deprivation following years of civil unrest which has left that part of the country with almost no health system. One of the first priorities undertaken with the assistance of the international donors following the signing of the Peace Accord has been to develop and initiate a programme to train midwives for the community. Elsewhere in Africa, new programmes for direct entry into midwifery training have just started, such as in Zambia.

In Bolivia, with UNFPA support, plans have been agreed and work commenced to introduce a pre-service midwifery programme, at provincial university level, so that the midwives from this programme will be educated to a level equivalent of other healthcare providers such as nurses. The reason behind the decision to start such a programme is that, despite excellent results of the national insurance scheme, many women are still reluctant to be attended to by a professional provider until a problem arises, often too late. This is because in the rural areas, where the majority of families still live, people feel that healthcare providers at the facility do not respect the cultural requirements surrounding pregnancy and childbirth. This new programme for professional midwives will have a large component on social and cultural issues, as well as on technical midwifery care. The work is being undertaken with technical support from Chile, which is one of the countries with the longest history of professional midwives in Latin America [81]. Haiti is also in the process of re-opening the national school of nursing and midwifery, after many years of deterioration of their health system due to internal conflict.

In many parts of Asia the same positive signs can also be observed. In 2006, Pakistan took the decision to mount a large initiative to train more than 58 000 community midwives. The first intake of students commenced in the summer of 2007. The competencies for this programme and the training of the midwife teachers were done in collaboration with and support from the ICM. The programme for introducing this new cadre has not taken a traditional vertical approach, but has started with strengthening the regulatory and accreditation system, through fortifying the Pakistan Nursing Council, establishing a new Midwifery Association (affiliated with the ICM), and working with the State Examinations Boards. The MOH supported by partners has also strengthened the training infrastructure, including upgrading and refurbishing training schools, as well as updating the staff working in the facilities where students will also undertake part of their training and where it is hoped they will refer clients after their graduation when needed. Afghanistan has recently re-opened their schools of midwives, after having started with launching a competency-based pre-service training curriculum. This successful programme allowed 1300 young midwives to graduate and make a dramatic impact on women's access to maternity care.

### 'Skilling up' and increasing retention of the current maternity workers

With the exceptions cited above, very few countries have embarked on a scheme for introducing a new cadre of professional midwife. Most countries in all regions have mainly focused on scaling up and skilling-up those who are already functioning as midwives, or supporting and retaining the midwifery providers working in isolated places. Mauritania for example is expanding an obstetric risk insurance mechanism aimed at sharing costs related to obstetric complications among all pregnant women on a voluntary basis. The budget includes a number of incentives (30%) and duty allowances (13%) to compensate facilities and staff for increased workload and is aimed at suppression of informal payments by clients. A mechanism of special incentives to ensure better retention of health professionals in remote areas has also been established, while noting that this initiative is not without its challenges, given the increasing competition from an uncontrolled and rapidly developing private sector [82].

Senegal on the other hand is focusing on strengthening management systems and capacity, especially at the district level. Professional staff are now receiving incentives and midwives, who are seen as the most cost-effective health professionals, are involved in maternal death reviews and focus groups to assist them in improving quality of care [83].

Rwanda is also undertaking a management approach to ensuring that skilled midwifery providers are available and accessible, free of charge and offering quality care. A recent survey has shown improvements in health centre performance and higher productivity of health staff through output-based performance contracts [84].

Mozambique, Malawi, Senegal, Tanzania and a few other African countries have for some years successfully trained mid-level cadres (health officers and midwives), as well as general practitioners to provide comprehensive emergency obstetric care including surgery. Their initial skills were deficient in terms of maternal and newborn health, and therefore as generalists they were unable to meet the needs of mothers and newborns. These trained health professionals are highly cost-effective as their training (and other related costs) is less costly in regard to the comparable performance of obstetric specialists. Furthermore, there is evidence showing their high level of retention [85,86].

### Expanding numbers of professional midwives to take services to the community

In Malawi, as in many African countries, professional midwives mainly conduct institutional births, yet the majority of births still take place at home. Also, like many neighbouring countries, Malawi suffers from a huge deficit of all human resources for health, including physicians, with a ratio of 1.6/100 000 (health workers/population). Addressing HRH challenges is very difficult, but action is being taken to expand training institutions to accommodate more students; increase enrolment of nurse/midwives and other healthcare providers; and to skill up competencies to gain community midwifery clinical experience. Moreover, a community-oriented curriculum has been developed to train District Health Officers, as a specific response to the huge numbers lost through migration. The programme includes a minimum of community health (25%), plus surgical and medical specialties, including midwifery skills. A post-graduate programme has now also been added [87].

Zimbabwe where maternal mortality increased between 1994 and 1999 from 283/100 000 to 695/100 000, is facing major challenges in relation to midwifery services, including high attrition rate (brain drain), inadequate midwife tutors, midwifery not seen as a lucrative post graduation career, and no recognition for the profession of midwifery. The curriculum has been revised, student midwives now have practical attachments (hands on experience), a new diploma in midwifery has been started, in-service training and on-the-job support (mentorship by a skilled midwife) are now standardized. Efforts to increase the capacities of training of teachers have resulted in development of a Masters with a major in Maternal and Child Health. WHO has also recently announced support for working in collaboration with the Royal College of Midwives (UK) to encourage some of the diaspora community who are in the UK working as midwife teachers to return for short stays to offer their services in Malawi.

In some countries the low rate of skilled attendance is not because there are insufficient providers, but because of insufficient posts in the public sector to employ all available healthcare providers, even if they are known to have the necessary skills. In Kenya, an initiative began in 2004 to explore if it were possible to empower retired midwives and to support them to return to work as semi-private practitioners, still linked to and supervised by the local facility, under authority of the District Management Team. This pilot project has proved to be highly effective in increasing the numbers of skilled midwifery providers working in the country – particularly at the community level, where almost half the births still take place. As in some other countries in the region, the age of retirement from public service is low in Kenya, currently 55 years of age. Many professional women, who have delayed their own pregnancies and childrearing until after they have completed their studies and have had little time to work, find this age too early. Many are still supporting children through higher education and out of necessity are required to keep earning an income. Although a formal evaluation of the initiative is not yet available, all stakeholders are enthusiastic with the preliminary results, and the MoH has now asked all donors to support this initiative. A decision has been made in the new national RH strategy to roll-out the programme across the country. One of the keys of the success of this initiative according to UNFPA and MoH has been the involvement of the community in selecting which retired midwives to support. Those that have been selected are valued in their respective communities and are being well used by the local families. Preliminary results show that referrals for complications have increased significantly particularly referrals from midwives who have been able to identify problems or potential problems early.

## Conclusion: Scaling up and skilling up

We hope to have conveyed the message that for the sake of mothers and newborns both 'scaling up' coverage and 'skilling up' quality of care are necessary. In the event of scarce resources, however, we support the option of giving priority to quality of care over coverage, offering an adequate number of skilled professionals strongly supported by a well performing system, rather than the option of a high number of multi-purpose workers based in villages without adequate capacity, authority and support. We do not believe, and the experience of Bangladesh and Indonesia seem to confirm, that a high number of community-based, multi-purpose workers can be properly supported and funded to achieve the desired objective. Also, our message is that even though specialist skilled professionals are preferable, they cannot, and should not, work alone. We introduce a fundamental contrast between 'community-midwives', who we consider unable to fulfill the core life-saving functions and 'midwives in the community', who are midwives first, with all the skills attached to the definition.

Overloading skilled professionals, particularly with tasks that can be done by others, is not cost-effective and can lead to burn out and poor quality. While 'multi-purpose community workers' can deliver other complementary services such as family planning and other primary health care services, it is not cost effective to produce multi-purpose workers with 'some' midwifery skills. Properly trained specialist skilled attendants such as professional midwives may take 3 to 4 years to train, they can have additional skills and deliver a broad range of primary healthcare, provided doing so does not interfere with the provision and maintenance of the competencies required to be a skilled birth attendant.

Developing the needed workforce to ensure that women and newborns have access to a competent midwifery provider requires a comprehensive plan, tailored to the specific situation in each country. We believe that the framework developed by the participants at the 1^st ^International Forum on Scaling up Midwifery for the Community can help countries to develop such a plan, while keeping a focus on quality [Additional file 1].

While countries should keep in mind from the beginning the 'long-term strategy' consisting of most births taking place in health centres (even small facilities operated by teams of midwives) attended by skilled professionals operating in multidisciplinary teams, and backed up by accessible functioning referral hospitals, their health planners also need to be pragmatic and to consider possible 'interim strategies'. An example of one such strategy is professional midwives leading multi-purpose teams and supervising home births attended by other health workers. However, there must be time limits set for these interim strategies otherwise they might become permanent strategies, as was the case in too many settings over the past 20 years.

Our final message is that monitoring and evaluation must be built into all plans from the very beginning, including for interim strategies, in an effort to produce evidence on how best to develop a competent midwifery workforce in low-resource settings. There must be a greater focus on continuous monitoring and periodic evaluations. Furthermore, monitoring and evaluation must focus on qualitative as well as quantitative data and look at the performance of providers – measuring how they are performing and identifying the system barriers that prevent quality performance.

## Competing interests

The authors declare that they have no competing interests.

## Authors' contributions

VF conceived the paper, drafted the outline, the research question and the conclusion, reviewed and edited the whole manuscript. DRS drafted the analysis of the current situation and the challenges, as well as the key areas of work to scale up midwifery. LdB drafted the chapter on lessons learned from countries. All three authors contributed to the reference search, read and approved the final manuscript.

## Supplementary Material

Additional file 1 The Hammammet Call to Action, 15 December 2006. The full text of the joint inter-agency declaration and call to action for governments, regulatory bodies, professional health care organizations, educators and communities. Resulting from the first International Forum on Midwifery in the Community in Hammammet, Tunisia Click here for file
