# Supplementary material for: Human resources for maternal health: multi-purpose or specialists?
Source: Hum Resour Health. 2008 Sep 30;6:21. doi: 10.1186/1478-4491-6-21 (PMC2569064; doi:10.1186/1478-4491-6-21)
Supplement: Additional file 1 — The Hammammet Call to Action, 15 December 2006. The full text of the joint inter-agency declaration and call to action for governments, regulatory bodies, professional health care organizations, educators and communities. Resulting from the first International Forum on Midwifery in the Community in Hammammet, Tunisia [file 1478-4491-6-21-S1.doc]

**Annex**

**The Hammammet Call to Action, 15 December, 2006**

The 1^st^ International Forum in Hammamet, Tunisia, on “Midwifery for the Community” gathered international agencies and organizations, along with midwives, nurses, physicians, health policy makers, professional associations, regulatory bodies and researchers from 24 countries around the world where maternal and neonatal mortality and morbidity remain unacceptably high. Having reviewed progress and constraints over these last twenty years, since the launch of the Global Safe Motherhood Initiative, participants concluded that intensified action is needed at global, regional and national levels to scale up the midwifery workforce needed to work in contact with communities. This midwifery workforce will help preventing most maternal deaths as well as post-delivery problems such as obstetric fistula, and help reducing the alarming rate of neonatal morbidity and mortality. In 2005 the World Health Organization estimated that 334,000 more midwives and others with midwifery skills are needed around the world. If well supported by effective health systems, midwives will help governments to achieve their fourth and fifth Millennium Development Goals by 2015.

The Call to Action says:

We, the participants of the 1^st^ International Forum on Midwifery for the Community, call on governments, regulatory bodies, professional health care organizations, educators, and communities worldwide to ensure the provision of midwifery services in the community by establishing or improving the following key areas:

-Policies to ensure equitable access to midwifery services;

-Policies and regulatory systems to improve the number, deployment, status and conditions of work of midwives and others with midwifery skills;

-Competency-based education and training in midwifery skills;

-Peer and supportive supervision of providers in the field;

-An enabling environment to support effective healthcare delivery, including infrastructure, communication, emergency transportation, adequate funding, equipment and supplies; and

-Permanent monitoring and periodic evaluation.

We believe that these actions will strengthen midwifery as an integrated part of the healthcare in the community. In addition they will improve the continuum of care needed to protect the health of women and babies, and save their lives by increasing access to emergency obstetric and neonatal care.

We also believe that it is the collective obligation of all stakeholders to guarantee mothers and their newborns their human rights to safe pregnancy, childbirth, and a safe postpartum recovery wherever they may live.
